# Effects of acute and sustained administration of vortioxetine on the serotonin system in the hippocampus: electrophysiological studies in the rat brain

**DOI:** 10.1007/s00213-015-3870-9

**Published:** 2015-02-11

**Authors:** Mostafa El Mansari, Maurice Lecours, Pierre Blier

**Affiliations:** University of Ottawa Institute of Mental Health Research, 1145 Carling Avenue, Ottawa, ON K1Z 7K4 Canada

**Keywords:** Electrophysiology, Serotonin, Hippocampus, Vortioxetine, Escitalopram, 5-HT_1A_ receptor, 5-HT_1B_ receptor

## Abstract

**Rationale:**

Vortioxetine is a novel multimodal antidepressant that is a 5-HT_1B_ receptor partial agonist, a 5-HT_1A_ receptor agonist, an inhibitor of the serotonin (5-HT) transporter, and a 5-HT_1D_, 5-HT_3_, and 5-HT_7_ receptor antagonist in vitro. In vivo studies have shown that vortioxetine enhances levels of 5-HT and desensitizes 5-HT_1A_ autoreceptors.

**Objectives:**

The aim of the present study was to investigate the effects of acute and long-term administration of vortioxetine on the terminal 5-HT_1B_ receptor and the tonic activation of 5-HT_1A_ receptor in the rat hippocampus.

**Methods:**

These receptors were assessed following vortioxetine administration acutely or subcutaneously using minipumps for 14 days. These studies were carried out using in vivo electrophysiological recording, microiontophoresis, and stimulation of the ascending 5-HT fibers.

**Results:**

Vortioxetine enhanced the inhibitory effect of the stimulation of the 5-HT bundle at a high, but not low frequency and reversed the inhibitory effect of the 5-HT_1B_ receptor agonist CP 94253. These results indicate that this compound acted as a 5-HT_1B_ receptor partial agonist. Vortioxetine inhibited 5-HT reuptake but did not dampen the sensitivity of postsynaptic 5-HT_1A_ receptors on pyramidal neurons. Long-term administration of vortioxetine and escitalopram (both at 5 mg/kg/day) induced an increase of tonic activation of the 5-HT_1A_ receptors in CA3 pyramidal neurons, resulting in an increase in 5-HT transmission. In addition, vortioxetine decreased the function of terminal 5-HT_1B_ autoreceptor following its sustained administration.

**Conclusions:**

Desensitization of 5-HT_1B_ autoreceptor and an increase of tonic activation of 5-HT_1A_ receptors in the hippocampus may contribute to the antidepressant effect of vortioxetine.

## Introduction

Clinical studies have established that vortioxetine is an effective antidepressant with a potential benefit on cognitive functions (Citrome 2014; McIntyre et al. 2013; Alvarez et al. 2014; Katona et al. 2012; Mahableshwarkar et al. 2014). In preclinical studies, vortioxetine displays two modes of pharmacological action on the serotonin (5-hydroxytryptamine, 5-HT) system: it selectively blocks the 5-HT transporter (5-HTT) and binds with moderate to high affinity to human (h) 5-HT_1A_, 5-HT_1B_, 5-HT_1D_, 5-HT_3_, and 5-HT_7_ receptors (Mørk et al. 2012). In vitro, it is a full agonist at 5-HT_1A_ receptors, a partial agonist at 5-HT_1B_ receptors, and an antagonist at 5-HT_1D_, 5-HT_3_, and 5-HT_7_ receptors.

A previous electrophysiological study showed that acute administration of vortioxetine potently suppresses the firing rate of dorsal raphe (DR) 5-HT neurons without optimally occupying the 5-HTT (Bétry et al. 2013). This is unlikely to result from a direct activation of the somatodendritic 5-HT_1A_ autoreceptors because vortioxetine has an affinity about 30 times lower for the rat (r)5-HT_1A_ receptor than for the 5-HTT (Mørk et al. 2012; Pehrson et al. 2013). Consequently, this marked suppression of firing may be due to the enhanced synaptic 5-HT availability resulting from synergy between 5-HTT inhibition and activation of receptor(s), as it is reversed by the selective 5-HT_1A_ receptor antagonist WAY-100635 (Bétry et al. 2013). Another action of vortioxetine on 5-HT neuronal firing distinguishes it from selective 5-HT reuptake inhibitors (SSRIs): during its sustained administration in rats, the (r)5-HT_1A_ autoreceptor is already desensitized and the firing rate of 5-HT neurons returns to control level within 3 days (Bétry et al. 2013). In contrast, the firing rate of 5-HT neurons returns to normal after 2 to 3 weeks of administration of SSRIs, which is also due to 5-HT_1A_ autoreceptor desensitization (Blier and de Montigny 1983; Czachura and Rasmussen 2000; El Mansari et al. 2005).

A unique property of vortioxetine is its affinity for 5-HT_1B_ receptors; in vitro, it appears to act as a partial agonist (Mørk et al. 2012). Such receptors are located on 5-HT terminals, where they exert an inhibitory action on 5-HT release, and postsynaptically as well. Importantly, in vitro and in vivo approaches have shown that the terminal 5-HT_1B_ autoreceptors desensitize following long-term administration of SSRIs (Blier and Bouchard 1994; Chaput et al. 1986, 1991; Dremencov et al. 2002). This adaptive change leads to a greater release of 5-HT for each impulse reaching 5-HT terminals, thus contributing to the enhancement of 5-HT transmission in the presence of SSRIs (Blier and El Mansari 2013).

The first goal of this study was to provide in vivo evidence for the partial agonistic action of vortioxetine on the terminal (r)5-HT_1B_ autoreceptor. The second was to determine whether vortioxetine could enhance 5-HT_1A_ transmission in the hippocampus using a regimen producing a suboptimal inhibition of the 5-HTT. Indeed, a 3-day administration of vortioxetine (5 mg/kg/day) resulted in only 41% occupancy of (r)5-HTT (Mørk et al. 2012). It was also shown that a daily dose of 10 mg of vortioxetine in humans is effective in major depressive disorder but only results in about 50% occupancy of 5-HTTs, in contrast to minimal effective doses of SSRIs, which produce about 80% 5-HTT occupancy (Areberg et al. 2012; Meyer et al. 2004; Stenkrona et al. 2013). Finally, this study was aimed at assessing the function of the terminal 5-HT_1B_ receptor following its long-term activation with vortioxetine.

## Materials and methods

### Animals 

Adult male Sprague–Dawley rats (Charles River, Saint-Constant, QC, Canada) weighing 250–350g at the time of the experiments were used. Animals were housed two per cage under standard laboratory conditions (12:12-h light/dark cycle; light cycle start at 7:00am; temperature 21 ± 1°C, 40–50% relative humidity) with access to food and water ad libitum. All animals were handled in accordance with the guidelines of the Canadian Council on Animal Care and the local animal care committee of the University of Ottawa Institute of Mental Health Research (Ottawa, Canada).

### Drug administration

Rats were anesthetized with isoflurane to implant the osmotic Alzet minipumps (Durect, Palo Alto, CA, USA), which delivered vehicle, vortioxetine subcutaneously at a dose of 5 mg/kg/day for 2 or 14 days, or escitalopram at a dose of 5 mg/kg/day for 14 days. These doses were selected on the basis of previous electrophysiological studies (Bétry et al. 2013).

### Extracellular recording and microiontophoresis of CA3 dorsal hippocampus pyramidal neurons

Rats were anesthetized with chloral hydrate (400 mg/kg, i.p.) and mounted in a stereotaxic apparatus. Supplemental doses of chloral hydrate (50-100 mg/kg, i.p.) were given to prevent any nociceptive reaction to tail or hind paw pinch. A burr hole was drilled at specific stereotaxic coordinates for the defined region for recordings, and neurons were identified by their spike shape, duration, and frequency. Neuronal activity was recorded in real time using the Spike2 software (Cambridge Electronic Design, Cambridge, UK), which was also used for analysis offline the electrophysiological characteristics of neurons. Body temperature was maintained at 37°C throughout the experiments using a thermistor-controlled heating pad. A catheter was inserted in the lateral tail vein for intravenous injections of pharmacological agents.

Extracellular recording and microiontophoresis were performed with five-barreled glass micropipettes in CA3 region of the hippocampus. The central barrel, used for the unitary recording, was filled with a 2 M NaCl solution, and the impedance of these electrodes ranged from 2 to 4 MΩ. The side barrels were filled with the following solutions: 5-HT creatinine sulfate (10 mM in 200 mM NaCl, pH 4), quisqualic acid (1.5 mM in 200 mM NaCl, pH 8), and the last barrel with a 2 M NaCl solution used for automatic current balancing. The micropipette was lowered into the dorsal hippocampus CA3 region using the following coordinates: 4mm anterior to lambda and 4.2 mm lateral (Paxinos and Watson 1998). A small current of quisqualate was used to activate the pyramidal neurons within their physiological firing range (10 to 15 Hz; Ranck 1973) because these neurons do not discharge spontaneously in chloral hydrate anesthetized rats. The hippocampus CA3 pyramidal neurons are found at a depth of 4.0 mm below the surface of the brain and identified by their large amplitude (0.5–1.2 mV) and long-duration (0.8–1.2 ms) simple action potentials, alternating with complex spike discharges (Kandel and Spencer 1961). 5-HT was microiontophoretically applied for 50-s periods with a current of 20 nA. The duration of local 5-HT application and ejection currents (nA) was kept constant before and after each intravenous (i.v.) injection of the 5-HT_1A_ receptor antagonist WAY-100635.

### In vivo determination of the sensitivity of 5-HT_1A_ receptors

In rat that received vehicle or vortioxetine, pyramidal neuron responsiveness to the microiontophoretic application of 5-HT was quantified by means of the IT50 index (nC), i.e., the current (nA) multiplied by the time (s) required to obtain 50% inhibition of spontaneous firing rate of pyramidal neurons (Brunel and de Montigny 1988).

### In vivo determination of 5-HT uptake

In order to assess the relative degree to which vortioxetine (5 mg/kg/day) and escitalopram (5 mg/kg/day) blocks the 5-HTT, the RT50 values were determined after microiontophoretic application of 5-HT (20 nA for 50 s) in hippocampus CA3 region. The RT50 values correspond to the time (s) elapsed from the cessation of microiontophoretic application of 5-HT to 50% recovery of the initial firing rate (Fig. 3; Pineyro et al. 1994). It is a reliable index of the 5-HT reuptake process in vivo. Indeed, previous experiments showed that administration of the SSRIs escitalopram and paroxetine (both 10 mg/kg/day) increased by threefold the RT50 values (El Mansari et al. 2005; Pineyro et al. 1994). Furthermore, the same degree of prolongation of the RT50 value was also observed in rats after lesion of 5-HT neurons, thereby eliminating 5-HTT (Pineyro et al. 1994).

### The 5-HT pathway stimulation

The CA3 region of hippocampus receives extensive projections from the median and DR 5-HT neurons. In order to electrically stimulate the ascending 5-HT pathway, a bipolar electrode (NE-100, David Kopf, Tujunga, CA, USA) was implanted 1mm anterior to lambda on the midline with a 10° backward angle in the ventromedial tegmentum and 8.0 ± 0.2mm below the surface of the brain. Two hundred square pulses with duration of 0.5 ms were delivered by a stimulator (S48, Grass Instruments, West Warwick, RI, USA) at an intensity of 300 μA and a frequency of 1 and 5 Hz. The stimulation of the 5-HT pathway induces a brief suppressant period due to the release of 5-HT into the synapse. The effects of stimulation of the ascending 5-HT pathway were assessed to determine the function of terminal 5-HT_1B_ autoreceptors (Chaput et al. 1986, 1991; Blier and de Montigny 1994). The two series of 300 stimulation pulses delivered at 1 and 5 Hz were carried out because previous studies showed that activation of terminal 5-HT_1B_ autoreceptors decreases 5-HT release in the terminal areas and that increasing the frequency of stimulation from 1 to 5 Hz induces a greater activation of 5-HT_1B_ autoreceptors, and consequently a greater negative feedback on the release of 5-HT (Chaput et al. 1986, 1991). As a result, the smaller release of the neurotransmitter in the synapse obtained at 5 Hz induces a shorter period of suppression compared to that obtained at 1 Hz. The stimulation pulses and the firing activity were analyzed by computer using Spike 2 software (Cambridge Electronic Design Limited, UK). Peristimulus time histograms (PSTH) of CA3 pyramidal neurons were generated to determine the suppression of firing measured as duration of suppression of firing (DOS in milliseconds). DOS is defined as the time interval initiated by a 50% reduction in the number of events per bin from the mean prestimulation probability of firing, to the time it returned to 90% of that same prestimulation value.

### Determination of the tonic activation of 5-HT_1A_ receptor in the hippocampus

In order to assess the degree of activation of the 5-HT_1A_ receptors exerting an inhibitory influence on the firing activity of CA_3_ pyramidal neurons, the selective 5-HT_1A_ receptor antagonist WAY-100635 was administered intravenously to disinhibit these neurons resulting in an increase of their firing activity (Haddjeri et al. 1998). The disinhibition would be best determined if the neurons were not firing at a high rate; therefore, their firing rate was decreased by reducing the ejection current of quisqualate, and after a baseline activity, the selective 5-HT_1A_ receptor antagonist WAY-100635 (25–100 μg/kg) was injected intravenously afterward. It can be assumed that any increment in the firing activity of hippocampus pyramidal neurons would reflect an increased level in the tonic activation of the 5-HT_1A_ receptors, and the degree to which WAY-100635 disinhibit this firing would be a direct measure of the tonic level of activation of these receptors by extracellular 5-HT. The percent change of firing activity was assessed by calculating the mean firing rate of neurons from about 2min prior to and after i.v. administrations of the drug.

### Drug injections

**Fig. 1:**
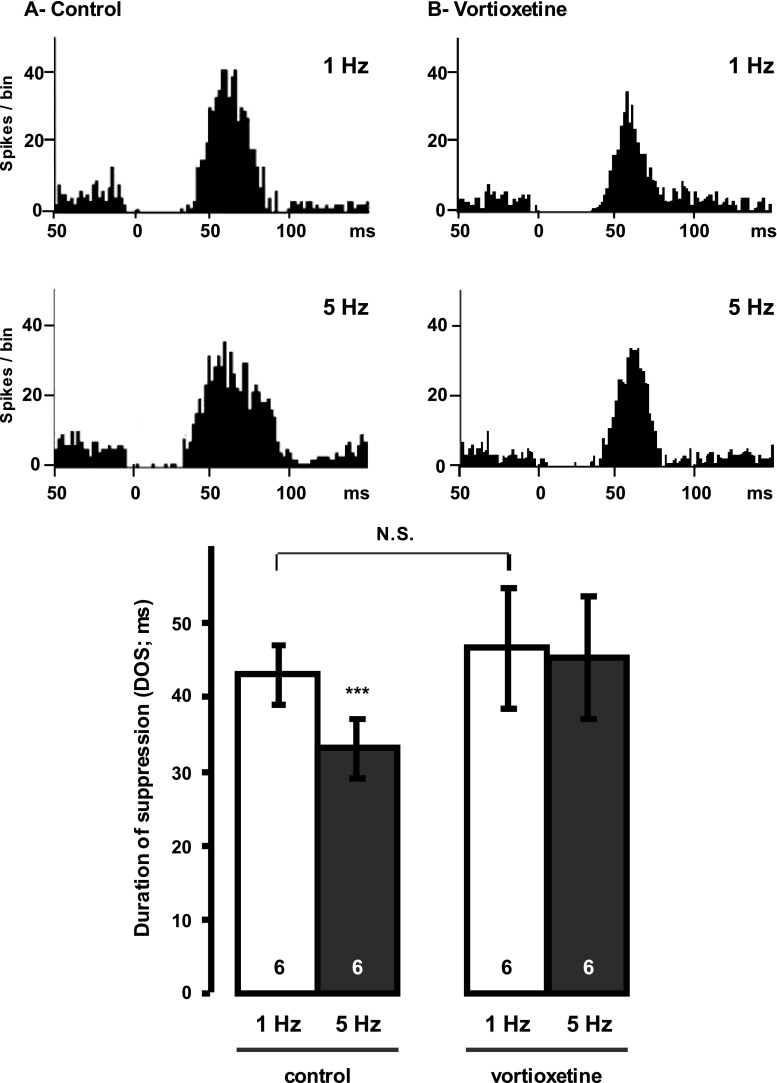
Assessment of the effect of vortioxetine by itself on terminal 5-HT_1B_ autoreceptors in the hippocampus. Peristimulus time histograms illustrating effects of stimulation (1 vs 5 Hz) of the ascending 5-HT pathway on the firing activity of CA3 pyramidal neurons before (control; **a**) and following the i.v. administration of vortioxetine (**b**; 6 mg/kg). Hence, after 1- and 5 Hz stimulations were completed, vortioxetine was injected followed at least 1 min after by 1 and 5 Hz stimulations in the same rat (*n* = 6). ***P < 0.001 compared to control; *N.S*. non-significant difference. *DOS* is the duration of suppression (ms) of the firing of pyramidal neurons induced by endogenous 5-HT following stimulation of the 5-HT bundle

**Fig. 2:**
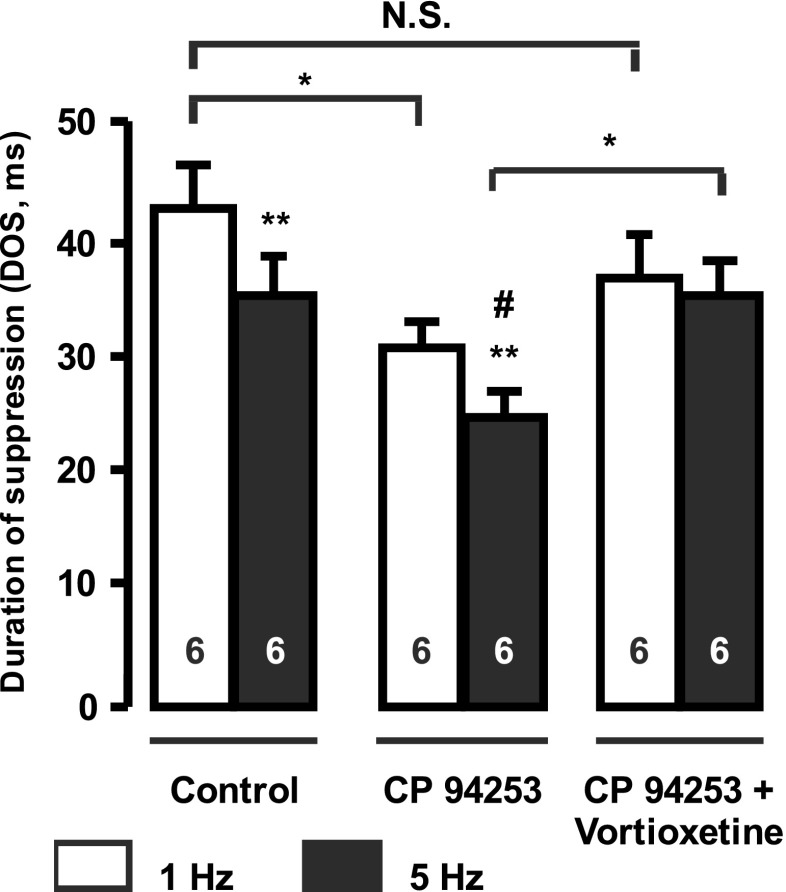
Assessment of the effect of vortioxetine (6 mg/kg) on the hippocampus terminal 5-HT_1B_ autoreceptor following its activation by the 5-HT_1B_ receptor agonist CP 94253 (2 mg/kg). Peristimulus time histograms showing effects of stimulation of the ascending 5-HT pathway (1 - 5 Hz) on the firing activity of pyramidal neuron in control, followed at least 1min after by i.v. injection of CP 94253 and then i.v. administration of vortioxetine on the same neuron. Only one neuron was tested per rat (*n* = 6). *P < 0.05; **P < 0.01; ***P < 0.001. *N.S.* non-significant difference. ^#^ *P* < 0.05, significant difference in DOS determined at 5 Hz following administration of CP 94253 when compared to controls. *DOS* is the duration of suppression (ms) of the firing of pyramidal neurons induced by endogenous 5-HT following stimulation of the 5-HT bundle

In the acute studies, every neuron was examined by the whole sequence of drugs (Figs. 1 and 2). Only one neuron was tested per rat to avoid residual effects. At least 1min lasted after drug i.v. injection before the start of stimulation.

### Drugs

Vortioxetine and escitalopram were provided by Lundbeck. WAY-100635, 5-HT creatinine sulfate, and chloral hydrate were purchased from Sigma (St. Louis, MO, USA). Quisqualic acid and CP 94253 were purchased from Tocris (Ellisville, MO, USA). Vortioxetine and CP 94253 were dissolved in 20% hydroxypropyl-beta-cyclodextrin. Escitalopram was dissolved in a 0.9% NaCl solution. WAY-100635 was dissolved in distilled water.

### Data analysis

The data are presented as mean values ± SEM. Statistical comparisons were carried out using a one-way or Kruskal-Wallis one-way ANOVA on ranks followed by Dunn’s method. Drug administration and stimulation (1 vs 5 Hz) were used as main factors, and statistical analyses of the data were done with two-way repeated measures analysis of variance (ANOVA), followed for all pairwise multiple comparisons by the Tukey LSD post hoc analysis. Statistical significance was taken as *P* < 0.05.

## Results

### Effect of the acute administration of vortioxetine on 5-HT_1B_ receptor activation in the hippocampus

In order to examine the net effect of vortioxetine on terminal 5-HT_1B_ autoreceptor activation by electrically evoked release of endogenous 5-HT, the effectiveness of two series of stimulations was delivered at different frequencies while recording from same pyramidal neuron. Indeed, the higher the frequency of stimulation is, greater should be the degree of activation of the terminal autoreceptor. Consequently, the smaller release of the neurotransmitter in the synapse obtained at 5 Hz induces a shorter period of suppression compared to that obtained at 1 Hz.

First, when the effect of vortioxetine per se was tested (Fig. 1), a two-way ANOVA with repeated measures showed a significant effect of the stimulation (F[1, 22] = 9.5; *P* < 0.05) and a significant interaction factor (stimulation × drug administration F[1, 22] = 9.2; *P* < 0.05). The Tukey post hoc test showed that in control, 5-HT-induced inhibition of pyramidal neurons was significantly decreased after stimulation with 5 Hz when compared to 1 Hz stimulation (*P* < 0.05; *n* = 6), whereas this inhibition was no longer statistically significant between 1 and 5 Hz, following i.v. administration of vortioxetine to the same rat (*P* > 0.05). Taken together, these results suggest that vortioxetine blocked the activation of 5-HT_1B_ receptors. It showed also that vortioxetine did not enhance the efficacy of the stimulation at 1 Hz when tested on same neuron (*P* > 0.05; Fig. 1), as a 5-HT_1B_ receptor antagonist such as methiotepin would normally do (Chaput et al. 1986), showing that vortioxetine is not a pure antagonist.

In the second series of experiments (Fig. 2), the effect of vortioxetine was tested following activation of the 5-HT_1B_ receptors by the potent and selective 5-HT_1B_ receptor agonist CP 94253. A two-way ANOVA with repeated measures on the inhibition resulting from endogenous 5-HT release following electrical stimulation revealed a significant effect of vortioxetine (F[2, 33] = 4.7; *P* < 0.05) and stimulation (F[2, 33] = 75.6; *P* < 0.001), as well as a significant interaction (stimulation × drug treatment; F[2, 33] = 6.4; *P* < 0.05). Using Tukey post hoc test and as previously reported in naive rats (Chaput et al. 1986, 1991), the 5-HT-induced inhibition of the firing activity of pyramidal neurons following 5 Hz stimulations was significantly shorter than with stimulation at 1 Hz (*P* < 0.001; *n* = 6; Fig. 2). This effect has been shown to be due to an increase in activation of the terminal 5-HT_1B_ receptor stemming from an increase in 5-HT release in its immediate biophase during stimulation at 5 Hz. Following i.v. administration of CP 94253 (2 mg/kg) to the same rat, the duration of the inhibition at both 1 and 5 Hz stimulations was significantly shorter compared to pre-injection condition (*P* < 0.05; *n* = 6; Fig. 2), indicating an overactivation of 5-HT_1B_ receptors by this agonist. When vortioxetine (6 mg/kg) was i.v. injected after CP 93253, there was no significant difference between the effects of stimulation with 1 and 5 Hz (*P* > 0.05; *n* = 6; Fig. 2). This suggests that while vortioxetine is able to reverse the effect of a potent 5-HT_1B_ receptor agonist, it acted as a 5-HT_1B_ receptor partial agonist.

### In vivo 5-HTT blockade properties of vortioxetine

**Fig. 3:**
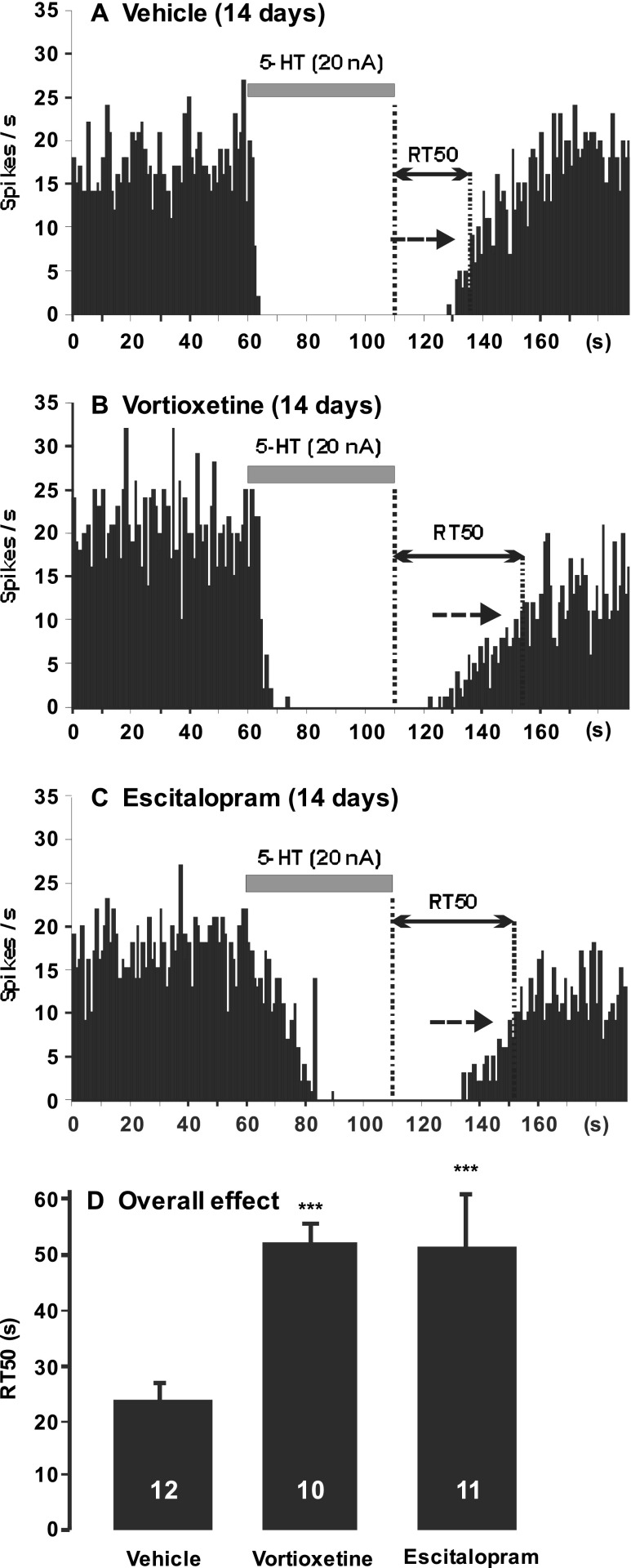
Integrated firing rate histograms of quisqualate (QUIS)-activated pyramidal neuron illustrating blockade of 5-HT transporters, as measured with RT50 index in rat that received vehicle (**a**; *n* = 12), vortioxetine (**b**; 5 mg/kg/day; *n* = 10), and escitalopram (**c**; 5 mg/kg/day; *n* = 11) for 14 days. The RT50 values correspond to the time in seconds elapsed from the cessation of microiontophoretic application of 5-HT (*horizontal gray bar*) to 50% recovery of the initial firing rate (indicated by a *dashed arrow*). **d** Note the increase in RT50 in rats that were administered with vortioxetine and escitalopram when compared to vehicle group. ***P < 0.001 compared to control

In order to measure reuptake inhibition by vortioxetine, the RT50 value was determined. Ejection of 5-HT at a current of 20 nA for 50s was kept constant and yielded an 80 to 100% inhibition of CA3 pyramidal neurons firing. Since the RT50 value is better determined when neurons are completely inhibited, only those responding with 100% inhibition were considered for RT50 analysis (Fig. 3). The mean RT50 for control rats administered with vehicle was 24 ± 3s (*n* = 12) and increased to 52 ± 4s in rats treated for 14 days with vortioxetine (*n* = 10) and to 51 ± 9s in rats that received a suboptimal dose of escitalopram for 14 days (*n* = 11; Kruskal-Wallis one-way ANOVA on ranks followed by Dunn’s method; *P* < 0.001 for both drugs compared to vehicle). The significant enhancement in the RT50 indicates a blockade of 5-HT reuptake transporters in the CA3 region of the hippocampus in rats that received vortioxetine or escitalopram..

### Sensitivity of 5-HT_1A_ receptors on pyramidal neurons of the CA3 region of the hippocampus

IT50 values were determined as a measure of postsynaptic 5-HT_1A_ receptor sensitivity. As shown in Fig. 3, there was no alteration in sensitivity of the 5-HT_1A_ receptors, when 5-HT was microiontophoretically applied at currents of 20 nA (IT50 values for control (*n* = 12), 48 nC ± 4; 14-day vortioxetine (*n* = 10), 60 nC ± 6; escitalopram (*n* = 8), 45 nC ± 7 (one-way ANOVA; *P* > 0.05)). This result showed that sensitivity of the 5-HT_1A_ receptors on CA3 pyramidal neurons was not changed after administration of these drugs.

### Effect of vortioxetine on the tonic activation of the 5-HT_1A_ receptor in the dorsal hippocampus

**Fig. 4:**
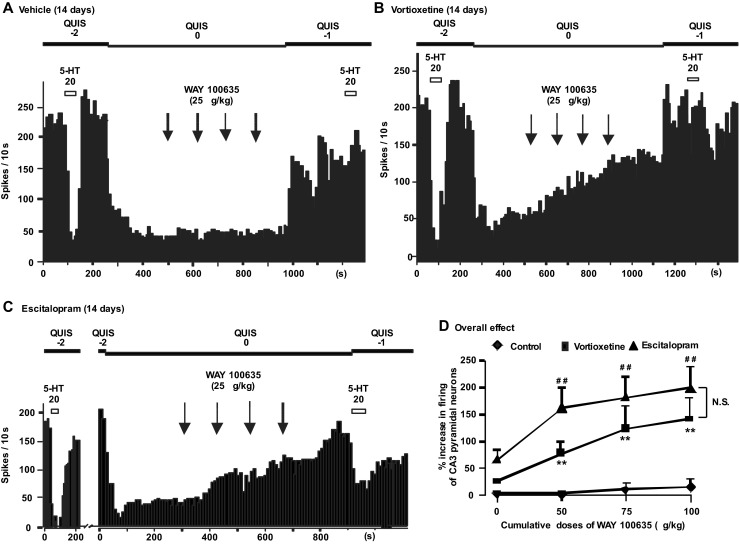
Integrated firing rate histograms of quisqualate (QUIS)-activated pyramidal neuron showing its responsiveness to the microiontophoretic application of 5-HT and i.v. injection of WAY-100635 in vehicle-treated rats (**a**; *n* = 8) and rats administered for 14 days with vortioxetine (**b**; 5 mg/kg/day; *n* = 6) and escitalopram (**c**; 5 mg/kg/day; *n* = 5). Note the increase in firing activity of pyramidal neurons after the injection of WAY-100635 (**b** and **c** compared to **a**), after which the inhibitory effect of 5-HT was blocked. **d** Degree (% ± SEM) of increase of the firing activity of pyramidal neurons after the administration of WAY-100635, in control rats and those administered with vortioxetine and escitalopram for 14 days. In each rat, only one neuron was tested. **P < 0.01 and ^##^ *P* < 0.01. *N.S.* non-significant difference

The effect of WAY-100635 on the quisqualate-activated firing activity of CA3 pyramidal neurons was assessed in rats that received vehicle, vortioxetine, and escitalopram for 14 days (Fig. 4). A two-way ANOVA (followed by Tukey post hoc test) on tonic activation of 5-HT_1A_ receptors in CA3 pyramidal neurons revealed a significant effect of drug treatment with vortioxetine and escitalopram ([F2, 73] = 12.9, *P <* 0.001), and WAY-100635 (F[2, 73] = 20.4, *P* < 0.001), and a significant interaction between both variables (F[2, 73] = 4.7, *P* < 0.001). Following sustained administration of vortioxetine and escitalopram, the tonic activation of the 5-HT_1A_ receptors by WAY-100635 was significantly enhanced compared to the vehicle group, reaching 141 and 197%, respectively, at a dose of 100μg/kg (Fig. 4; vehicle group, *n* = 8; vortioxetine group, *n* = 6; escitalopram group, *n* = 5). However, there was no significant difference between the vortioxetine and escitalopram groups.

### Assessment of the sensitivity of terminal 5-HT_1B_ autoreceptors

**Fig. 5:**
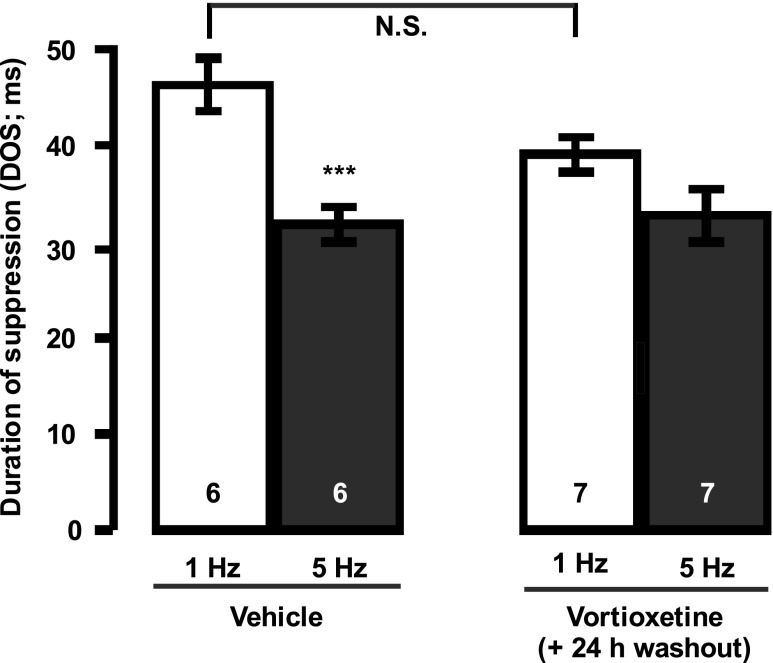
Effect of sustained administration of vortioxetine (5 mg/kg/day) on the responsiveness of terminal 5-HT_1B_ autoreceptors in the hippocampus. Peristimulus time histograms illustrating effects of stimulation of the ascending 5-HT pathway on the firing activity of CA3 pyramidal neurons in vehicle rats that received hydroxypropyl-beta-cyclodextrin (*n* = 6) and those that received vortioxetine for 14 days (*n* = 7), followed by a 24-h period of washout (to ascertain that the autoreceptor is desensitized and not blocked). *DOS* is the duration of suppression of the firing of pyramidal neurons induced by endogenous 5-HT following 5-HT bundle stimulation. Only one neuron was tested in each vehicle- or vortioxetine-administered rat. ***P < 0.001. *N.S.* non-significant difference

In order to determine if long-term administration of vortioxetine altered 5-HT_1B_ receptor responsiveness, rats were administered vehicle and vortioxetine for 14 days and electrical stimulation of the ascending 5-HT bundle was preformed (Fig. 5). Since vortioxetine has a strong affinity for the 5-HT_1B_ receptor, a decreased efficacy of electrical stimulation could be explained by either desensitization of the 5-HT_1B_ autoreceptor or competition between endogenous 5-HT and vortioxetine. For this reason, a 24-h washout period (considering a plasma elimination half-life of only 3.2-h; Mørk et al. 2012) was used to discount the second explanation of decreased electrical stimulation efficacy. Although the effect on 5-HT-induced inhibition of pyramidal neurons was not statistically significant following 14 days of vortioxetine administration (two-way ANOVA with repeated measures; F[1, 23] = 0.7; *P* > 0.05), the goal in these experiments was to determine if the decrease usually observed in control group following stimulations with 5 Hz is still present in the vortioxetine-treated animals. Interestingly, a significant effect of interaction (*P* < 0.05; F[1, 21] = 5.1; two-way ANOVA with repeated measures) and stimulation (*P* < 0.05; F[1, 21] = 24.6) was revealed following Tukey post hoc test. It showed that 5-HT-induced inhibition of pyramidal neurons was significantly decreased after stimulation with 5 Hz when compared to 1 Hz in vehicle-treated rats (*P* < 0.05; *n* = 6; Tukey post hoc test) and that this difference is no longer statistically significant between 1 and 5 Hz in rats that received vortioxetine for 14 days (*P* > 0.05; *n* = 7; Tukey post hoc test). This indicates that 14-day administration of vortioxetine resulted in desensitization of terminal 5-HT_1B_ autoreceptors in the hippocampus.

## Discussion

The present study showed that vortioxetine acted as a 5-HT_1B_ receptor partial agonist because it competed with both an exogenous 5-HT_1B_ receptor agonist and endogenous 5-HT under high but not low degree of activation of the terminal 5-HT_1B_ autoreceptor. It also showed that vortioxetine increased tonic activation of 5-HT_1A_ receptors on pyramidal neurons in the hippocampus resulting from enhanced 5-HT levels, because there was no change of the sensitivity of these postsynaptic 5-HT_1A_ receptors. Long-term vortioxetine administration also produced a desensitization of 5-HT_1B_ autoreceptors located on 5-HT terminals in the hippocampus.

Acute administration of vortioxetine had no effect on 5-HT-induced inhibition when the ascending 5-HT bundle was stimulated at a frequency of 1 Hz. If vortioxetine was acting as a pure 5-HT_1B_ receptor agonist, it should have reduced the suppression duration of firing as was found with the 5-HT_1B_ receptor agonist CP 94253. Had vortioxetine been acting as a pure antagonist, this inhibition would have at least doubled at 1 Hz stimulation, as was previously shown with the 5-HT_1B_ receptor antagonist methiotepin (Chaput et al. 1986). However, after the acute administration of vortioxetine and the overactivation of 5-HT_1B_ autoreceptors using 5-Hz stimulations, the decrease in inhibition was no longer significant (Fig. 1). Therefore, these results indicate that vortioxetine was acting as a 5-HT_1B_ receptor partial agonist, since it only competed with high levels of endogenous 5-HT, resulting from the higher frequency of stimulation. This property of vortioxetine was also reported in recombinant cell lines which showed that vortioxetine acts as a partial agonist at 5-HT_1B_ receptors (Mørk et al. 2012). Moreover, vortioxetine displays a partial agonistic activity at the h5-HT_1B_ receptor when measured in whole cell based cAMP assay (Mørk et al. 2012). It is noteworthy that the above mentioned acute effects of vortioxetine were not due to its 5-HT reuptake blocking property. Indeed, a previous study showed that acute administration of the SSRI citalopram, which completely suppresses the firing activity of 5-HT neurons, resulted in no alteration of the suppression of firing activity of CA3 pyramidal neurons from stimulation of the ascending 5-HT pathway (Chaput et al. 1986).

The present study confirmed previous results showing that the effect of iontophoresed 5-HT on pyramidal neurons is mainly via 5-HT_1A_ receptors as this effect was reversed by the selective 5-HT_1A_ receptor antagonist WAY-100635 (Fig. 4; El Mansari et al. 2005; Haddjeri et al. 1998). As reported herein, the 14-day administration of vortioxetine did not significantly change the sensitivity of 5-HT_1A_ receptors on CA3 pyramidal neurons, in line with previous studies with SSRIs (El Mansari et al. 2005) and 5-HT_1A_ receptor agonist such as gepirone (Haddjeri et al. 1998). It is worth noting that these 5-HT_1A_ receptors have nevertheless the capacity to desensitize as was shown following sustained monoamine oxidase A inhibition using the antidepressant clorgyline (Blier et al. 1986).

The current experiments were deemed to have been carried out in the presence of suboptimal 5-HTT inhibition. Indeed, the RT50 values obtained with both vortioxetine and escitalopram (both at 5 mg/kg/day, s.c.) administered by osmotic minipumps were doubled compared to controls. However, several reports from our group have shown that this index of in vivo reuptake is tripled in rats with lesioned 5-HT neurons to eliminate 5-HTT (Pineyro et al. 1994), and with regimens of 10 mg/kg/day of either escitalopram or paroxetine (El Mansari et al. 2005; Pineyro et al. 1994). Interestingly, the latter regimen of escitalopram produces in rats plasma levels similar to those observed in humans using therapeutic doses (10–20 mg/day; Bourke et al. 2013).

In the present study, a 14-day administration of vortioxetine and escitalopram (both at 5 mg/kg/day) produced an enhancement in the tonic activation of the 5-HT_1A_ receptor that is typical of SSRIs tested (see Blier and El Mansari 2013). Since the responsiveness of postsynaptic 5-HT_1A_ receptors on pyramidal neurons was unaltered, this enhancement must therefore be attributable to an elevation of synaptic 5-HT in their vicinity, as vortioxetine shows a very weak affinity for 5-HT_1A_ receptors in rats when compared to humans (Bang-Andersen et al. 2011; Mørk et al. 2012). Interestingly, in microdialysis studies, subchronic (3 days) administration of vortioxetine at this same dose (5 mg/kg/day) significantly elevated extracellular levels of 5-HT in ventral hippocampus (Mørk et al. 2012), although only 41% of 5-HTT was occupied when this dose is used. Interestingly, vortioxetine administration resulted in an increase in tonic activation of the 5-HT_1A_ receptors in the hippocampus that was similar to that seen with suboptimal dose of escitalopram, as shown in the present study. Furthermore, the effect of vortioxetine on tonic activation of 5-HT_1A_ receptors may be underestimated because, unlike in humans, this compound possesses a low affinity for 5-HT_1A_ and 5-HT_7_ receptors in rats (Bang-Andersen et al. 2011; Mørk et al. 2012). This is all the more important since previous studies showed that 5-HT_1A_ receptor agonists (e.g., gepirone; Haddjeri et al. 1998) and the 5-HT_7_ receptor antagonist SB269970 (Mnie-Filali et al. 2011) by increasing 5-HT transmission (Wesołowska et al. 2006) induced a robust enhancement of the tonic activation of 5-HT_1A_ receptors in the hippocampus (Blier and El Mansari 2013).

This increase in 5-HT transmission can be due, at least in part, to the attenuated function of the terminal 5-HT_1B_ autoreceptor. Indeed, the present results showed that following sustained administration of vortioxetine, this autoreceptor appeared to be desensitized since the difference in the efficacy of the 1 and 5 Hz stimulation was also no longer significant after a vortioxetine washout. In in vivo and brain slice experiments, long-term administration of various SSRIs was shown to induce a desensitization of the terminal 5-HT_1B_ autoreceptors that account for the increased effectiveness of 5-HT synaptic transmission in the rat hippocampus (Blier and Bouchard 1994; Chaput et al. 1986, 1991; Moret and Briley 1990). Previous studies showed that the concomitant blockade of SERT and 5-HT_1B_ receptors increases the extracellular 5-HT levels in the hippocampus (de Groote et al. 2003). Moreover, the selective 5-HT_1B/1D_ receptor antagonist GR127935, which by itself did not alter basal 5-HT levels, doubled them in animals when 5-HTT was blocked using fluoxetine or paroxetine (Gobert et al. 1997; Sharp et al. 1997). Interestingly, vortioxetine was also shown to increase levels of 5-HT more in the ventral hippocampus than in the medial prefrontal cortex (Pehrson et al. 2013) most likely by acting on several receptors, rather than just blocking the 5-HTT (Mørk et al. 2012; Pehrson et al. 2013).

In conclusion, vortioxetine blocks the 5-HTT but does not dampen the sensitivity of postsynaptic 5-HT_1A_ receptors. Long-term vortioxetine administration increases the tonic activation of the postsynaptic 5-HT_1A_ receptor in the hippocampus, an effect common to all antidepressants studied strategies so far. In addition, vortioxetine decreased the function of the terminal 5-HT_1B_ autoreceptor under a high but not a low degree of activation, thus showing that it acts as a partial agonist. This study has shown that vortioxetine exerts several different actions on the serotonergic system in the hippocampus, which can in part explain the action of vortioxetine as an effective antidepressant in the clinic (Alvarez et al. 2014). Furthermore, to these properties, previous preclinical studies have shown that vortioxetine is endowed with many properties that were shown to be involved in antidepressant action. Hence, vortioxetine is a 5-HT_7_ receptor antagonist, an h5-HT_1A_ receptor agonist, and a 5-HT_3_ receptor antagonist (Mørk et al. 2012; see also Pehrson and Sanchez 2014). Vortioxetine also showed antidepressant and anxiolytic-like effects in behavioral models (Guilloux et al. 2013).
